# Feasibility and safety of exercise during chemotherapy in people with gastrointestinal cancers: a pilot study

**DOI:** 10.1007/s00520-023-08017-6

**Published:** 2023-09-05

**Authors:** Song Ee Park, Du Hwan Kim, Don-Kyu Kim, Joo Young Ha, Joung-Soon Jang, Jin Hwa Choi, In Gyu Hwang

**Affiliations:** 1 Department of Internal Medicine, Chung-Ang University Gwang-Myeong Hospital, Chung-Ang University College of Medicine, Seoul, Korea; 2 Chung-Ang University Integrated Oncology and Palliative Care Research Institute, Seoul, Korea; 3 Department of Rehabilitation Medicine, Chung-Ang University College of Medicine, Seoul, Korea; 4 Department of Rehabilitation Medicine, Chung-Ang University Gwang-Myeong Hospital, Chung-Ang University College of Medicine, Seoul, Korea; 5 Department of Internal Medicine, Veterans Health Service Medical Center, Seoul, Korea; 6 Department of Internal Medicine, Uijeongbu Eulji Medical Center, Eulji University, Uijeongbu Gyuonggido, Korea; 7 Department of Radiation Oncology, Chung-Ang University College of Medicine, 84 Heukseok-Ro, Dongjak-Gu, Seoul, 06973 South Korea; 8 Department of Internal Medicine, Chung-Ang University College of Medicine, 84 Heukseok-Ro, Dongjak-Gu, Seoul, 06973 South Korea

**Keywords:** Cancer, Exercise, Chemotherapy, Overall survival

## Abstract

**Purpose:**

Sarcopenia is a poor prognostic factor in cancer patients, and exercise is one of the treatments to improve sarcopenia. However, there is currently insufficient evidence on whether exercise can improve sarcopenia in patients with advanced cancers. This study examined the feasibility of exercise in advanced gastrointestinal (GI) cancer patients treated with palliative chemotherapy.

**Methods:**

Between 2020 and 2021, 30 patients were enrolled in a resistance and aerobic exercise program for six weeks. The exercise intervention program (EIP) consisted of low, moderate, and high intensity levels. Patients were asked to select the intensity level according to their ability. The primary endpoint was the feasibility of the EIP measured by compliance during the six weeks. A compliance of over 50% was considered acceptable. The secondary endpoints were changes in weight and muscle mass, safety, quality of life (QoL) and overall survival (OS).

**Results:**

The median age of the study’s participants was 60 (30–77). The total compliance to the EIP was 63.3% (19/30 patients). Sixteen (53.3%) patients had a compliance of over 80%. The attrition rate was 30.0% (9/30). The mean exercise time was 41.4 min, and the aerobic exercise was 92.3% and the resistant exercise was 73.7%, and both exercise was 66.5%. Most patients performed the moderate intensity level exercises at home or near their home. The mean skeletal muscle index (SMI) was 43.5 cm^2^/m^2^ pre-chemotherapy and 42.2 cm^2^/m^2^ after six weeks of chemotherapy, with a decrease of -1.2 ± 2.8 cm^2^/m^2^ (-3.0%) *(p* = 0.030). In the poor compliance group, the mean SMI decrease was -2.8 ± 3.0 cm^2^/m^2^ which was significantly different *(p* = 0.033); however, in the good compliance group, the mean SMI decrease was -0.5 ± 2.5 cm^2^/m^2^ which was maintained over the six weeks *(p* = 0.337). The good compliance group had a significantly longer median OS compared with the poor compliance group (25.3 months vs. 7.9 months, HR = 0.306, 95% CI = 0.120–0.784, *p* = 0.014). The QoL showed a better score for insomnia (p = 0.042). There were no serious adverse events.

**Conclusions:**

The EIP during palliative chemotherapy in advanced GI cancer patients showed good compliance. In the good compliance group, muscle mass and physical functions were maintained for six weeks. The EIP was safe, and the QoL was maintained. Based on this study, further research in exercise intervention in advanced cancer patients is needed.

**Clinical trial registration:**

The clinical trial registration number is KCT 0005615 (CRIS, https://cris.nih.go.kr/cris/en/); registration date, 23rd Nov 2020.

**Supplementary Information:**

The online version contains supplementary material available at 10.1007/s00520-023-08017-6.

## Introduction

The loss of skeletal muscle, sarcopenia, has an exceptionally high prevalence in cancer patients, particularly in gastrointestinal (GI) cancers. The prevalence of sarcopenia in cancer patients before starting treatment is about 15–74% [1], and in particular, advanced GI cancer accounts for 47–60% of sarcopenia cancer cases [2–4]. Sarcopenia is a poor prognostic factor which adversely affects the overall survival (OS) rate of colorectal cancer patients who received adjuvant chemotherapy [4, 5].

Sarcopenia cannot be fully restored by traditional nutritional support [6]. It is crucial to start sarcopenia treatments at the optimal time, which has been reported to be at the time of cytotoxic chemotherapy, meaning that cancer sarcopenia interventions should be provided concurrently to the cytotoxic chemotherapy.

The exercise therapy approach has the advantage of reducing muscle loss due to chemotherapy and has been used to treat sarcopenia in several types of cancer. Mizrahi et al. reported an enrollment rate of 63% and an adherence rate of 81% after recurrent ovarian cancer patients receiving chemotherapy completed a 12-week exercise therapy program. There was a statistical improvement in the quality of life, fatigue, and muscle strength [7]. Ligibel et al. presented a randomized clinical trial with metastatic breast cancer patients who received 16 weeks of exercise therapy. Only 42% of patients received palliative chemotherapy in the exercise therapy group. There was no statistical improvement in the physical fitness of the treatment group [8].

Cancer patients have poor appetites, nausea and neutropenia due to chemotherapy and then they cannot exercise regularly. Cancer patients receiving cytotoxic chemotherapy may also have reduced muscle mass [9, 10]. We expected to improve chemotherapy-associated muscle weakness via aerobic and resistance exercise in cancer patients during chemotherapy.

The purpose of this study was to determine the compliance and attrition rate and the safety of an exercise treatment program in GI cancer patients during palliative chemotherapy. The results of this study can be used in planning future phase II trials since this study provides a reasonable estimate of the length, intensity and places of an exercise intervention program for patients undergoing chemotherapy.

## Methods

### Study design, recruitment, and eligibility

This study was conducted on GI cancer patients receiving palliative chemotherapy to estimate the compliance, safety, and efficacy of an exercise intervention program (EIP). The clinical trial registration number is KCT 0005615 (CRIS, https://cris.nih.go.kr/cris/en/). The protocol for this pilot and feasibility study was approved by the Institutional review board of Chung-Ang University Hospital (IRB No 2022–007-410).

We aimed to recruit 30 patients to be able to estimate the compliance, safety, and efficacy of an EIP during palliative chemotherapy. Eligible participants were 19 years of age or older; diagnosed with recurrent or metastatic gastric, colon, biliary, or pancreatic cancer; had an Eastern Cooperative Oncology Group (ECOG) score between 0 and 1; a life expectancy of more than three months. Patients with decreased cognitive function, exercise restrictions, or fractures were excluded from this study. All participating patients provided written informed consent and permission from their physicians to participate.

### Exercise interventions

This pilot study consisted of a 12-week concurrent EIP and palliative chemotherapy. All patients were required to perform an EIP for 6 weeks, and patients who comply with this program were allowed to maintain the exercise for an additional 6 weeks. After screening, eligible patients began receiving palliative chemotherapy and started an EIP. Patients received fourth to sixth cycles of chemotherapy with the same regimen. Before starting palliative chemotherapy, patients consulted the Department of Rehabilitation Medicine to conduct a baseline assessment and to receive education on the EIP.

The EIP consisted of three levels of exercise intensities: low, moderate, and high. Patients were asked to select the intensity according to their ability. Patients were recommended to start with the low intensity and to increase the intensity if tolerable. Each of the three levels of exercise intensity consisted of a total of 30–40 min. The low-intensity level recommended a total exercise time of 30 min with a warm-up exercise of 5 min, a main exercise of 20 min, and a cool-down exercise of 5 min. The moderate- to high-intensity level recommended a total exercise time of 40 min with a warm-up exercise of 5 min, a main exercise of 30 min, and a cool-down exercise of 5 min. The main exercise consisted of aerobic exercises such as walking on a treadmill, level walking or running for 15 min and resistant exercises using dumbbells, isotonic resistance bands or bicycle ergometer for 15 min. The low-intensity level was equivalent to 9–10 points on the Borg’s ratings of perceived exertion (PRE) and a heart rate reserve (HRR) of 30% during the main exercise. The moderate-intensity level was equivalent to 11–13 points on the PRE and an HRR of 40–50% during the main exercise. The high-intensity level was equivalent to 14–15 points on the PRE and an HRR of 70% during the main exercise.

### Measurements

The baseline assessment included blood tests, enhanced abdominopelvic computed tomography (APCT), hand grip strength, 6-m universal gait speed, and bioelectrical impedance analysis (InBody) and was performed before the EIP. Blood tests, including hemoglobin (Hb), total leukocytes count (TLC), albumin, prealbumin, transferrin, and c-reactive protein (CRP), were conducted at 6 and 12 weeks during the EIP. In addition, the APCT, handgrip strength, 6-m universal gait speed, and InBody measurements were evaluated at 6 and 12 weeks during the EIP.

The EIP was performed during the six weeks of palliative chemotherapy. Before the EIP, the rehabilitation department evaluated the exercise ability as a baseline assessment to confirm the exercise performance ability and measured the effectiveness variable for comparison after six weeks. The patient performed the EIP for six weeks. If the patient experienced no problems, the EIP was extended for 12 weeks, and the EIP was continuously evaluated for an additional second evaluation.

Quality of life was assessed using the European Organization for Research and Treatment of Cancer Quality of Life Questionnaire (EORTC-QLQ C30). The EORTC-QLQ C30 was given to patients as a baseline before the EIP, at 6 weeks after the EIP, and at 12 weeks after the EIP.

### Endpoints

The primary endpoints were compliance and attrition rates for the EIP. Compliance was assessed according to the thresholds of less than 50%, 50–80%, and over 80%. A compliance of more than 50% of exercise intervention time was considered acceptable. When a patient performed more than 50% of the suggested exercise time, it was defined that the patient had performed one exercise session. During the six weeks, exercises were performed five days a week, and if a patient performed 30 exercises sessions in total, it was determined that the patient had a compliance rate of 100%. The secondary endpoints were assessment of the skeletal muscle index (SMI) via CT and Inbody, body weight, body mass index (BMI), hand grip strength, and a six-minute walking test (6MWT). These were assessed at the baseline and after six weeks of the EIP. Safety and quality of life (QoL), progression-free survival (PFS), and OS were also assessed as secondary endpoints.

Skeletal muscle area was measured using an APCT. We evaluated SMI using the third lumbar vertebra (L3) muscle area, one of the international standards for measuring the ratio of skeletal muscle area (cm^2^) divided by height (m^2^). Sarcopenia is defined as a ratio less than 31 cm^2^/m^2^ for females and less than 49 cm^2^/m^2^ for males on the L3 skeletal index using the Korean-specific range [2].

### Statistical analysis

Statistical analyses were performed using the Statistical Package for Social Sciences (SPSS) version 24.0 (IBM Corp., Armonk, NY, USA). Statistical significance was defined as a *p*-value less than 0.05. A one-way ANOVA was used to analyze the mean exercise times. The number of patients who performed aerobic exercise and resistance exercise was compared using an independent *t*-test. The Kaplan–Meier method was used to estimate the survivals. Hazard ratios (HR) and their corresponding 95% confidence intervals (CI) were stratified using the Cox proportional hazards regression model.

## Results

### Patients

From November 2020 through August 2021, 30 patients with advanced gastrointestinal cancer undergoing palliative chemotherapy were recruited for this study. The baseline characteristics of these patients are shown in Table 1. The median age was 60 (30–77). Among them, 10 (33.3%) patients were over 65, and 17 (56.7%) were male. Nine patients (34.6%) had sarcopenia at the time of diagnosis, and 21 patients (70.0%) had a normal BMI.

**Table 1:** Baseline characteristics

Characteristics	Total
	(*n* = 30)
Age—year	
median	60
range	30–77
Age ≥ 65	10 (33.3%)
Sex, *n* (%)	
Male	17 (56.7%)
Height (cm), mean ± SD	162.0 ± 8.2
Weight (kg), mean ± SD	58.8 ± 10.6
BMI, kg/m^2^, mean ± SD	22.2 ± 3.1
Underweight < 20	6 (20.0%)
Normal 20–24.9	21 (70.0%)
Overweight > 25	3 (10.0%)
L3 Skeletal muscle index (cm^2^/m^2^)	43.5 ± 6.5
Sarcopenia, n (%) (total evaluation 26)	9 (34.6%)
Primary cancer	
Stomach cancer	3 (10.0%)
Esophageal cancer	3 (10.0%)
Colorectal cancer	11 (36.6%)
Biliary tract cancer	6 (20.0%)
Pancreas cancer	7 (23.4%)
Palliative chemotherapy line	
1^st^ line	26 (86.7%)
2^nd^ line	3 (10.0%)
3^rd^ line	1 (3.3%)

*Sarcopenia* was defined as an L3 skeletal muscle index of < 49 cm^2^/m^2^ for males and < 31 cm^2^/m^2^ for females using Korean-specific cutoffs

*SD* standard deviation, *BMI* body mass index

### Compliance

**Fig. 1:**
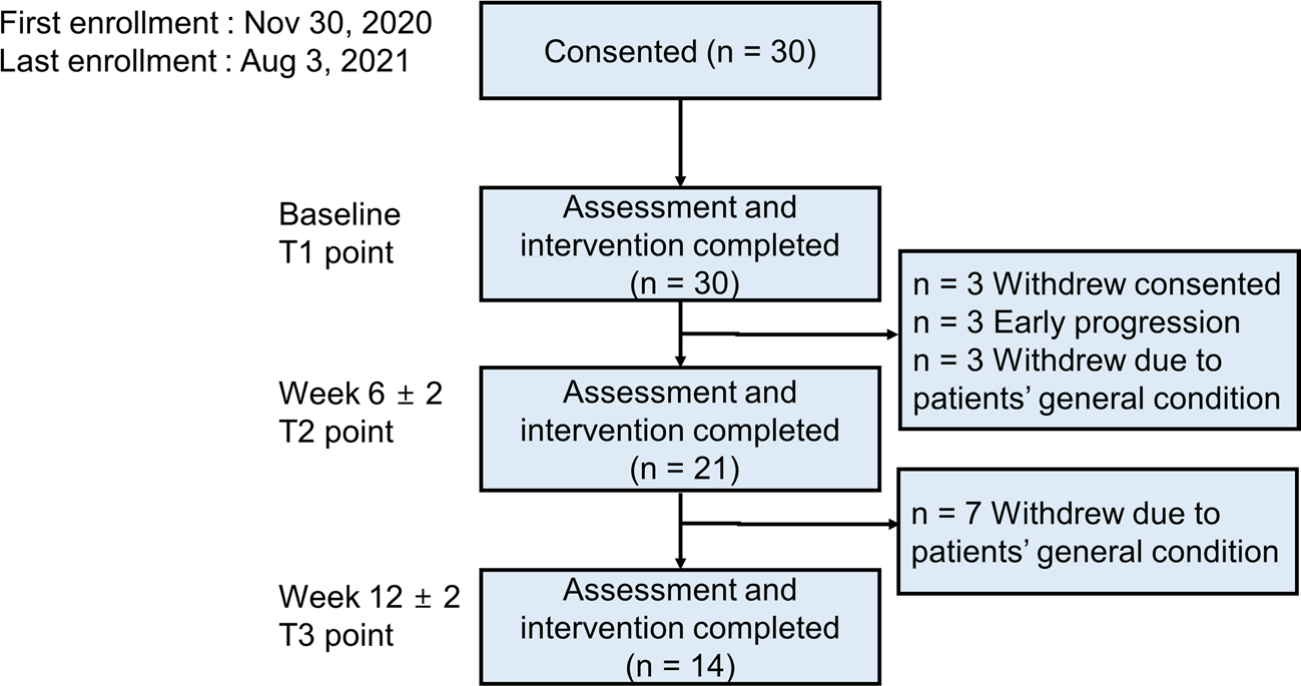
Patient flowchart

Thirty patients were analyzed at the start of the study (baseline T1 point) (Fig. 1). Three patients withdrew consent due to deterioration of their condition, and they failed to be assessed Inbody, hand grip test, and 6- meter wait gait test at baseline. Three patients had early progression before six weeks, and chemotherapy was stopped. Three patients could not complete the exercise due to their aggravated condition. The attrition rate was 30.0% (9/30). Exercise compliance rate was 63.3% (19/30) during the six weeks. Over the six weeks, the median number of total exercises session was 24. The mean time per session was 41.4 ± 38.80 min. Sixteen (53.3%) patients did over 80% of the resistance and aerobic exercises. The 27 patients who completed the baseline assessment were categorized into two groups, the good compliance group (*n* = 19) with a compliance of over 50% and the poor compliance group (*n* = 8) with a compliance of under 50%. The patients maintained EIP for 12 weeks was 46.7% (14/30).

### Characteristics of exercise in cancer patients

**Fig. 2:**
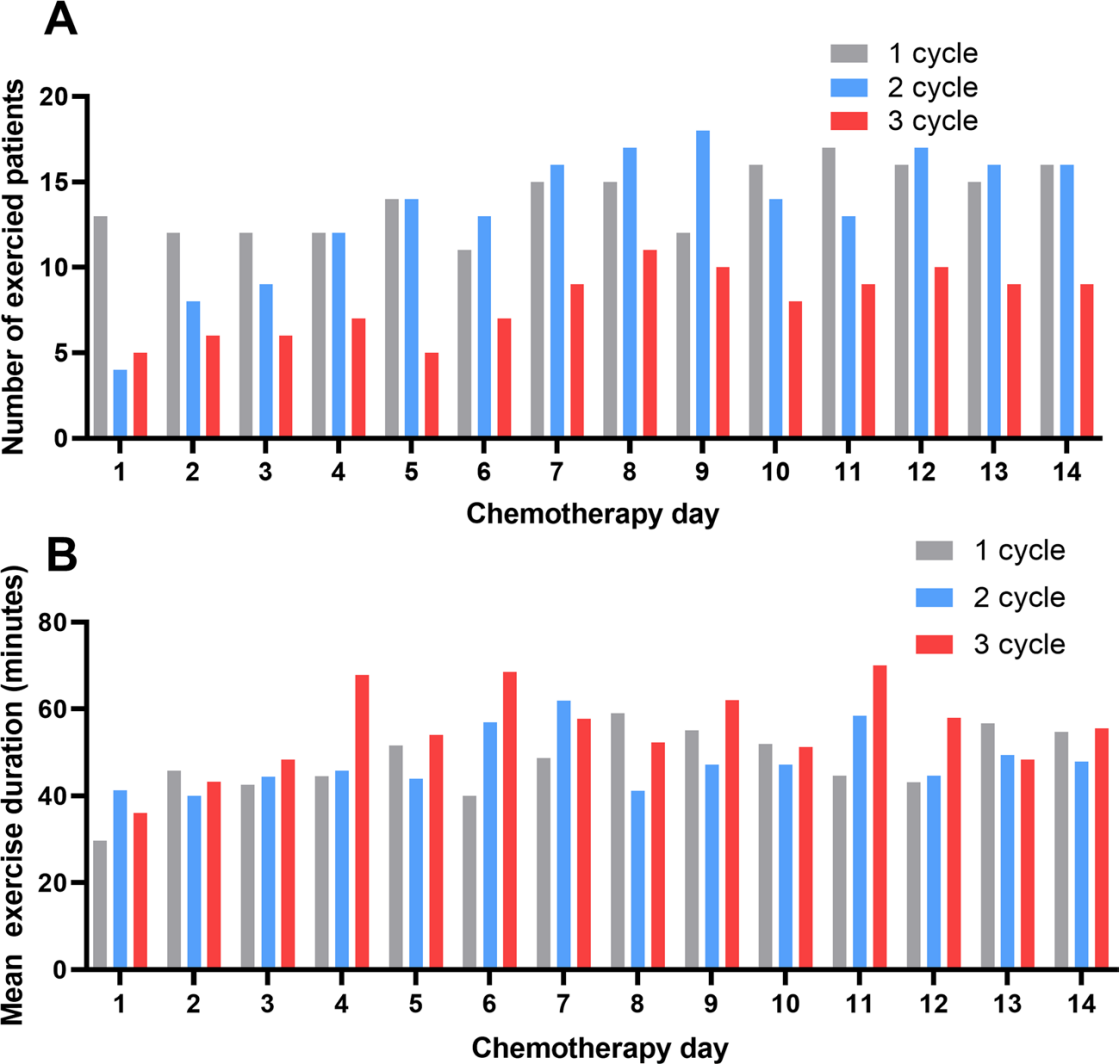
Characteristics of exercise in patients undergoing exercise treatment. **A** The number of patients who exercised and the chemotherapy day according to the cycle, **B** The mean exercise time and the chemotherapy day according to the cycle

The mean number of patients who exercised in first cycle was similar to mean number of patients in the second cycle (13.9 patients vs. 13.3 patients) (Fig. 2A). During the third cycle, fewer patients performed the exercises compared with first and second cycles (8.5 patients vs. 13.9, 13.3 patients). The mean exercise time was 41.4 min. The mean exercise time for cycles one, two, and three was 47.7 min, 47.8 min, and 55.2 min, respectively. The mean exercise time significantly increased in the third cycle of chemotherapy compared with the first and second cycles (*p* = 0.030) (Fig. 2B). The aerobic exercise was 92.3% and the resistant exercise was 73.7%, and both exercise was 66.5%. These results indicated that more patients completed the aerobics exercises than the resistance exercises (*p* = 0.005) (Fig. S1).

**Fig. 3:**
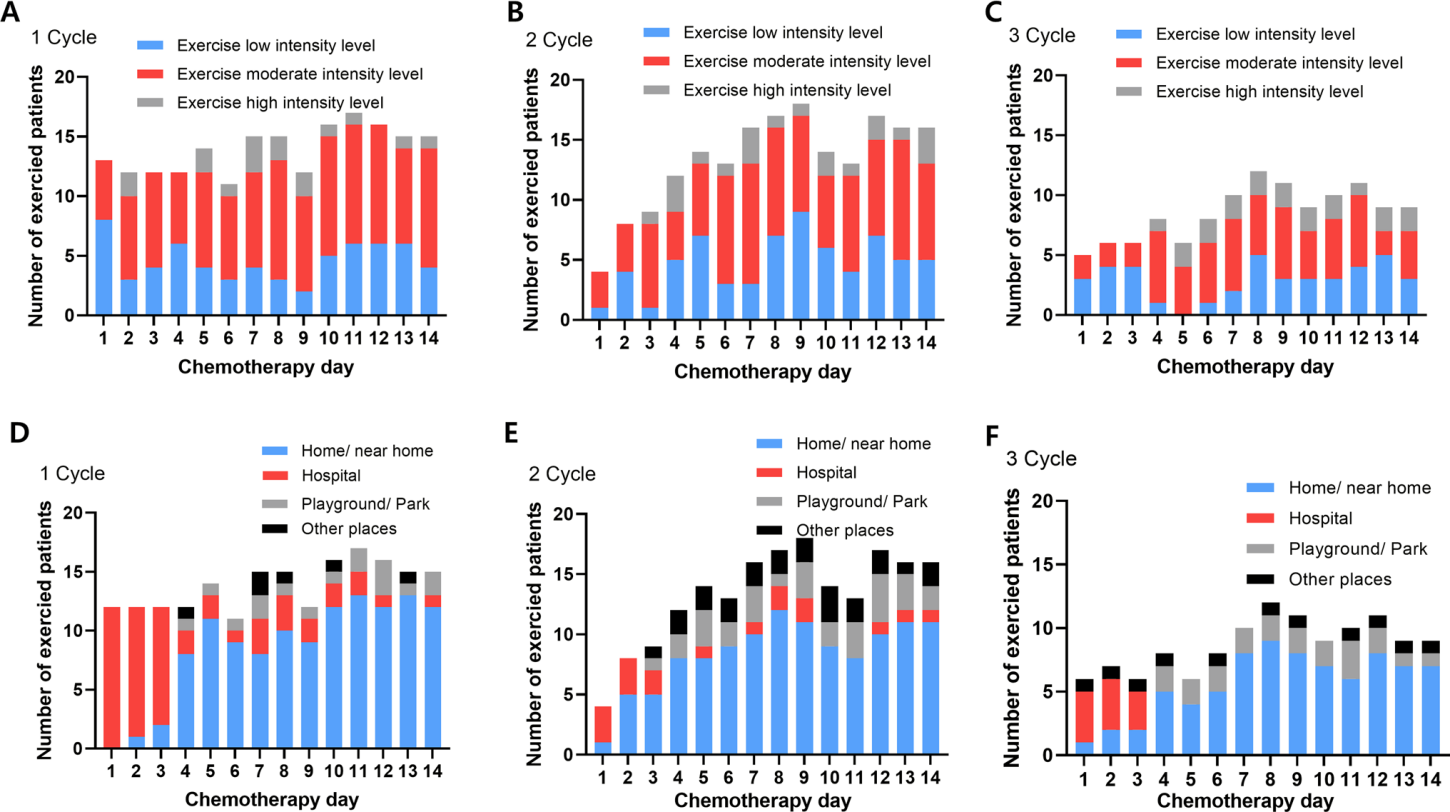
Exercise intensity and location The exercise intensity during (**A**) first cycle, (**B**) second cycle, and (**C**) third cycle. The exercise location during (**D**) first cycle, (**B**) second cycle, and (**C**) third cycle

During first, second and third cycles, 56.6%, 55.1%, and 48.7% of the patients performed the moderate-intensity exercises, respectively (Fig. 3A, B, C). During chemotherapy days one to three, most patients performed the exercises at the hospital. After chemotherapy day four, 60.0% of the patients performed the exercises at home or near their home (Fig. 3D, E, F).

### Body composition values and physical functions change according to exercise intervention program

**Fig. 4:**
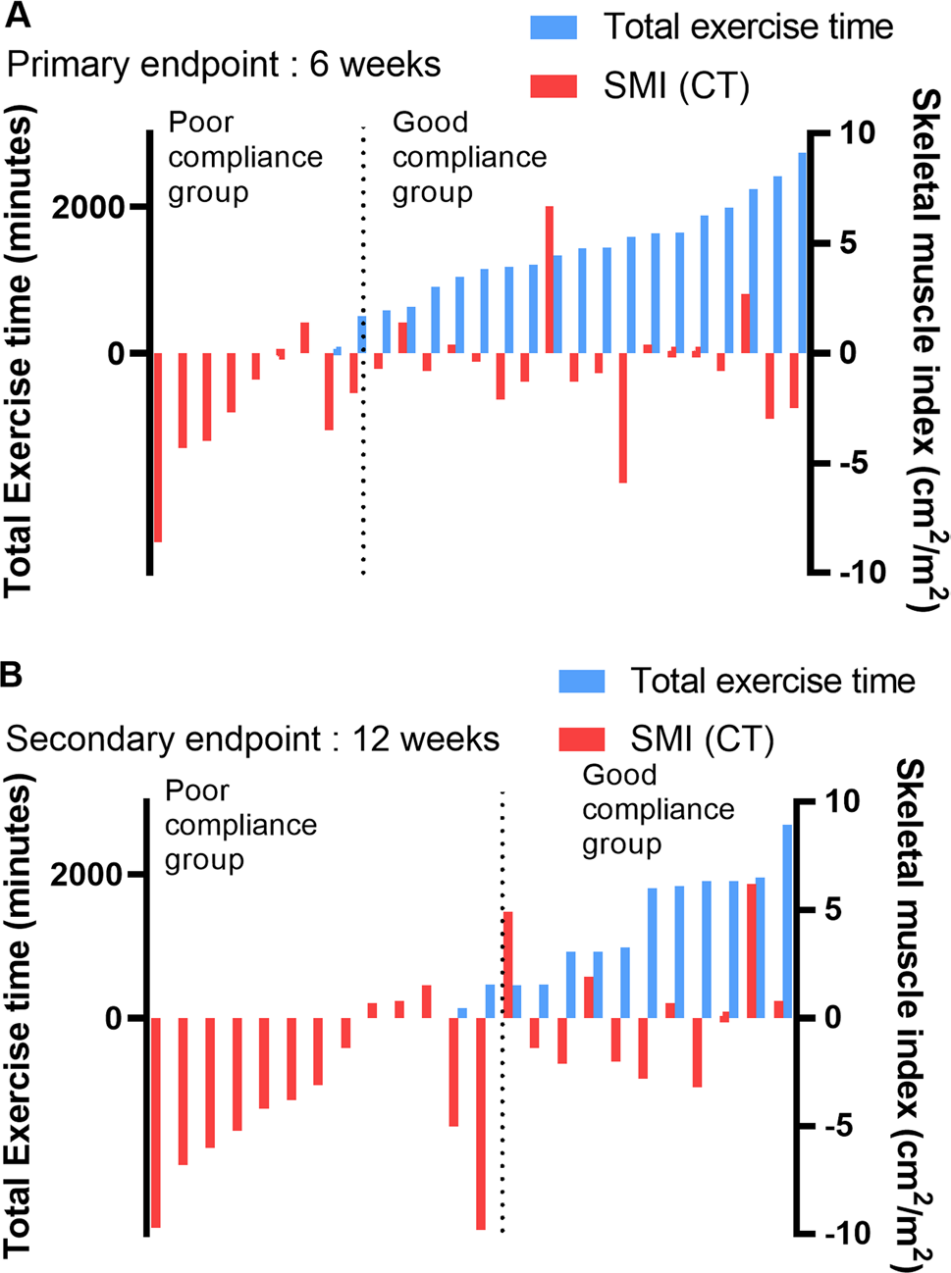
Correlation between total exercise time for six weeks and changes in the skeletal muscle index measured by CT. **A** 6-week evaluation (T2 point) (*n* = 27) and **B** 12-week evaluation (T3 point) (*n* = 21)

The mean body weight was 59.0 kg pre-chemotherapy and 59.4 kg after six weeks of chemotherapy (*p* = 0.716) (Table 2). The adjusted difference in mean total body lean mass (Inbody) after 12 weeks (-0.07 kg, *p* = 0.914) was not significant. The mean BMI was 22.0 kg/m^2^ pre-chemotherapy and 22.3 kg/m^2^ after six weeks of chemotherapy (*p* = 0.448). The mean SMI measure by CT was 43.5 cm^2^/m^2^ pre-chemotherapy and 42.7 cm^2^/m^2^ after six weeks of chemotherapy, with a mean decrease of -1.29 ± 2.86 cm^2^/m^2^ (-3.0%) (*p* = 0.030). After six weeks of chemotherapy, the poor compliance group’s mean SMI decreased by -2.8 ± 3.0 cm^2^/m^2^, which was significantly different (*p* = 0.033). The good compliance group’s mean SMI decreased by -0.5 ± 2.5 cm^2^/m^2^ and was maintained over the six weeks (*p* = 0.337) (Fig. 4A). The mean SMI measure by CT was 41.4 ± 7.9 cm^2^/m^2^ after 12 weeks of chemotherapy, with a mean decrease of -2.06 ± 3.90 cm^2^/m^2^ (-4.9%) (*p* = 0.014). After 12 weeks of chemotherapy, the poor compliance group’s mean SMI decreased by -0.3.5 ± 2.5 cm^2^/m^2^ and was significantly different (*p* = 0.005). The good compliance group’s mean SMI decreased by -1.3 ± 4.2 cm^2^/m^2^ and was maintained during the 12 weeks (*p* = 0.210) (Fig. 4B). There were no notable differences in the 6MWT and hand grip strength (Table 2).

**Table 2:** Body composition values and physical function change

	Baseline (*n* = 30)		6 weeks (*n* = 21)		12 weeks (*n* = 14)		Mean change over 6 weeks			Mean change over 12 weeks		
Measure	Mean	SD	Mean	SD	Mean	SD	Mean	95% CI	*p*-value	Mean	95% CI	*p*-value
Body weight (kg)	58.8	10.6	59.4	9.7	58.7	9.8	0.36	-1.68 to 2.41	0.716	-0.08	-2.12 to 1.95	0.929
BMI (kg/m^2^)	22.2	3.1	22.3	3.1	22.6	4.3	0.30	-0.51 to 1.12	0.448	0.02	-0.87 to 0.91	0.859
Skeletal muscle index (cm^2^/m^2^) (CT)	42.7	7.0	42.7	6.4	41.4	7.9	-1.29	-2.44 to -0.13	0.030	-2.06	-3.67 to 0.45	0.014
Skeletal muscle mass (Inbody)	24.8*	5.7*	24.6	5.4	23.1	4.9	-0.25	- 1.16 to 0.65	0.564	-0.17	-1.13 to 0.79	0.708
Fat mass, kg (Inbody)	13.5*	6*	14	5.2	15.9	7.5	0.88	-0.10 to 1.87	0.077	-0.05	-1.86 to 1.74	0.947
Lean body mass, Total body (Inbody)	42.6*	8.8*	42.5	8.3	40.3	7.7	-0.10	-1.60 to 1.20	0.809	-0.07	-1.48 to 1.33	0.914
Hand grip test	23.1*	8.1*	22.2	6.4	20.9	5.8	-0.24	-2.53 to 2.03	0.819	0.08	-2.24 to 2.40	0.938
6 m gait test	6.7*	2.6*	6.2	1.3	5.8	0.8	0.14	-0.66 to 0.95	0.704	-0.66	-2.30 to 0.98	0.372

*Missing data for Inbody, Hand grip test, and 6-m gait test due to patient’s poor general condition (*n* = 3, baseline)

*BMI* body mass index, *SD* standard deviation

### Survival

**Fig. 5:**
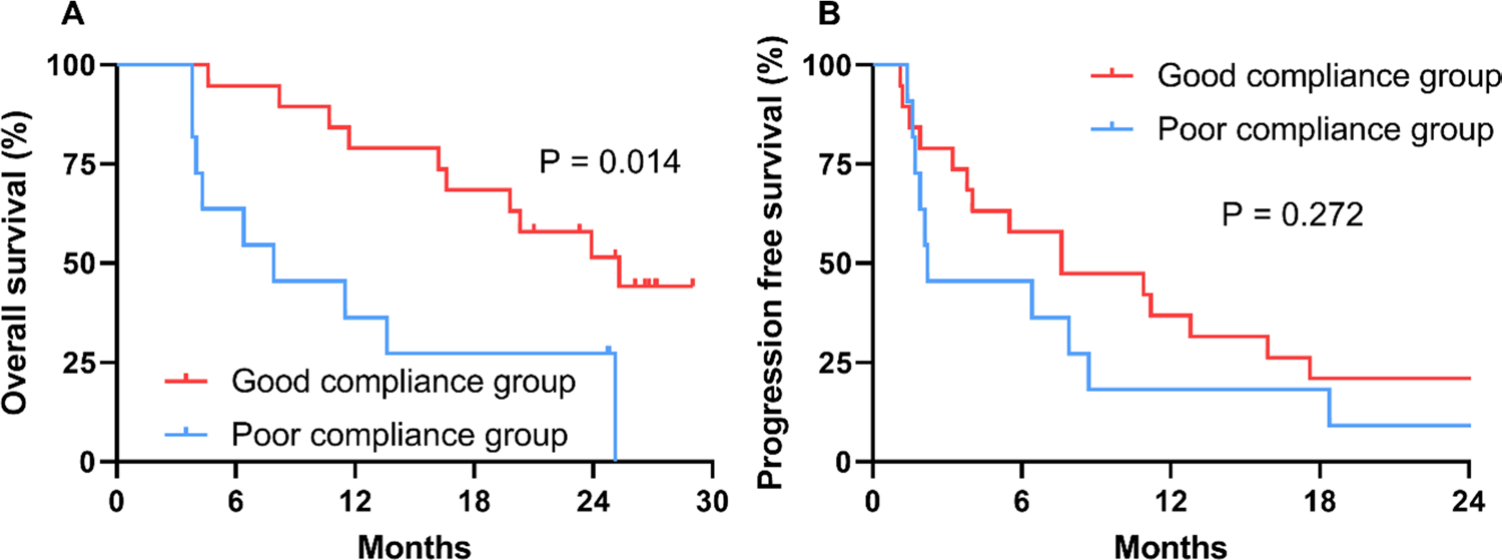
Survival outcomes according to the exercise compliance group. **A** Overall survival and **B** progression-free survival

The cutoff time for the analyses was April 2023 and resulted in a median follow-up of 26.6 months (3.8–29.0 months), and six patients (20.0%) died over this period. The median OS was 19.8 months (95% CI = 9.8–29.7 months). The median PFS was 6.4 months (95% CI = 1.5–11.2 months). The good compliance group had a significantly longer median OS compared with the poor compliance group (25.3 months vs. 7.9 months, HR = 0.306, 95% CI = 0.120–0.784, *p* = 0.014) (Fig. 5A). The median PFS of good compliance group was numerically better than the poor compliance group, but there was no statistical difference (7.6 months vs.2.2 months, HR = 0.636, 95% CI = 0.284–1.427, *p* = 0.272) (Fig. 5B). Multivariable analyses showed that the good compliance group was an independent prognostic indicator for OS (HR = 0.202, 95% CI = 0.046–0.878, *p* = 0.033) (Table S1).

### Blood biomarker

There were no differences in hemoglobin, albumin, prealbumin, TLC, transferrin saturation, and CRP during the six weeks of palliative chemotherapy and EIP. There was no difference in the blood biomarkers measured after 12 weeks of EIP (Table S2).

### Quality of life and adverse event

**Fig. 6:**
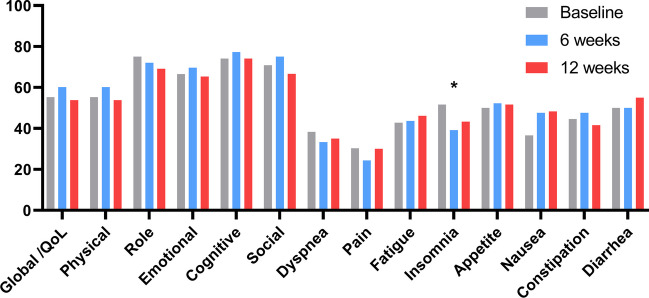
EORTC QLQ-C30 values and changes over exercise training

The quality of life assessed via the EORTC QLQ-C30 showed a better score for insomnia (*p* = 0.042) (Fig. 6). Although there was no statistically significant difference observed in the comparison of QoL between baseline and 6 weeks, there was a tendency for emotional, cognitive, and social quality of life to improve (Table S3). At the end of the 6th week, two patients had Grade 2 anemia toxicity, and at the end of the 12th week, four patients had Grade 2 anemia. There were no serious adverse events during the testing or EIP.

## Discussion

This study provided valuable data concerning the feasibility and safety of an EIP during palliative chemotherapy in patients with advanced GI cancers. In patients with advanced GI cancers treated with cytotoxic chemotherapy, 63.3% of patients exercised during the six weeks. The mean exercise time was 41.4 min. Most patients mainly performed aerobic and moderate-intensity exercises at home or near home.

We were able to successfully recruit advanced GI cancer patients that were receiving palliative chemotherapy. In another study, Solhemi et al. reported a randomized phase II study on multimodal intervention in patients with advanced non-small cell and pancreatic cancers [11]. The compliance rate for exercise training was only 60%, which was similar to the 63.3% in this study. In this study, poor compliance group who participated in the EIP did not exercise after returning home. Therefore, it is important to perform follow-ups with the patients in the EIP to increase the compliance. Also, regular phone calls and promotion of the EIP through social networking services (SNS) could increase compliance.

Cancer patients undergo multiple cycles of chemotherapy, and as the cycle number increased, the number of patients who exercised decreased. Although the number of patients who performed the exercises was small, those who exercised during the third cycle significantly increased their mean exercise time to 55 min. An EIP during palliative chemotherapy may be helpful for patients accustomed to exercising during chemotherapy.

Aerobic exercises were performed more than resistant exercises, as expected by the researchers. Although many patients only did aerobic exercises, there was also a proportion that did both aerobic and resistant exercises. Moderate-intensity exercise was mainly performed at the patient’s house or near their home except for exercising at the hospital while hospitalized. We were concerned about the safety of performed resistant exercise and high intensity exercise during chemotherapy. However, some patients were able to safely perform resistant and high-intensity EIP program.

This feasibility study had three other outcomes. First, there was a significant decrease in the overall SMI in the enrolled patients, measured by CT. However, the SMI in the good compliance group was not significantly decreased. Second, the good compliance group had significantly improved OS compared with the poor compliance group. Third, the quality of life of patients that exercised showed improvements in insomnia.

Compared with other papers, the advantage of this paper is that patients with GI cancer received concurrent palliative chemotherapy and EIP. Previous studies showed that when patients with GI cancers received palliative chemotherapy, the SMI measured by CT significantly decreased after six weeks of chemotherapy, and the mean SMI decreased by – 5.3 cm^2^/m^2^ [3]. The SMI significantly decreased in the total patients undergoing EIP in this study, but the mean SMI decrease was slight compared with the previous study’s results (-0.1 cm^2^/m^2^ vs. -5.3 cm^2^m/^2^). In the Nutrition and Exercise Treatment for Advanced Cancer (NEXTAC) study, exercise therapy was administered for eight weeks during chemotherapy in lung and pancreatic cancer patients, and the decrease in mean SMI was -1.1 cm^2^/m^2^, similar to this study [12].

In a previous study, the decreased SMI group had significantly shorter OS rates [3]. Therefore, this study was performed to test if the median OS was improved by reducing the decrease in the SMI by performing an EIP at the same time as chemotherapy. In the good compliance group, the SMI did not significantly decrease during the EIP period. Accordingly, it suggested that EIP during palliative chemotherapy cannot prevent the decrease in SMI. However, it was helpful in reducing the decrease in the SMI, and the decrease in SMI was prevented when the exercise compliance was good (≥ 50%).

In another previous study, CRP and albumin were measured, and there were no differences between the control and exercise groups [11]. This study measured blood biomarkers that were expected to be relevant three times, at the baseline, 6 weeks, and 12 weeks, but the blood biomarkers showed no differences between the groups during palliative chemotherapy.

However, through this study, we suggested that sarcopenia was still occurring during chemotherapy, even with an EIP. Therefore, we have planned a further study to overcome this limitation. The authors are currently conducting a study on an exercise and nutrition combination therapy to prevent the decreased in SMI for older cancer patients during palliative chemotherapy.

In conclusion, an EIP during palliative chemotherapy in advanced GI cancer patients had a good compliance. In the good compliance group, muscle mass and physical functions were maintained for six weeks. The exercise intervention was safe, and the quality of life was maintained. The good compliance group had significantly improved OS compared with the poor compliance group. Based on this study, further research is needed.

### Supplementary Information

Below is the link to the electronic supplementary material.

Supplementary file1 (DOCX 206 KB)

Supplementary file2 (DOCX 15 KB)

Supplementary file3 (DOCX 14 KB)

Supplementary file4 (DOCX 15 KB)

## Data Availability

The data presented in this study are available on request from the corresponding author.
