# Supplementary material for: Feasibility and safety of exercise during chemotherapy in people with gastrointestinal cancers: a pilot study
Source: Support Care Cancer. 2023 Sep 5;31(10):561. doi: 10.1007/s00520-023-08017-6 (PMC10480261; doi:10.1007/s00520-023-08017-6)

**Figure S1.** The number of patients who exercised according to the type of exercise and the chemotherapy day during six weeks; 1^st^ cycle (A), 2^nd^ cycle (B) and 3^rd^ cycle (C).


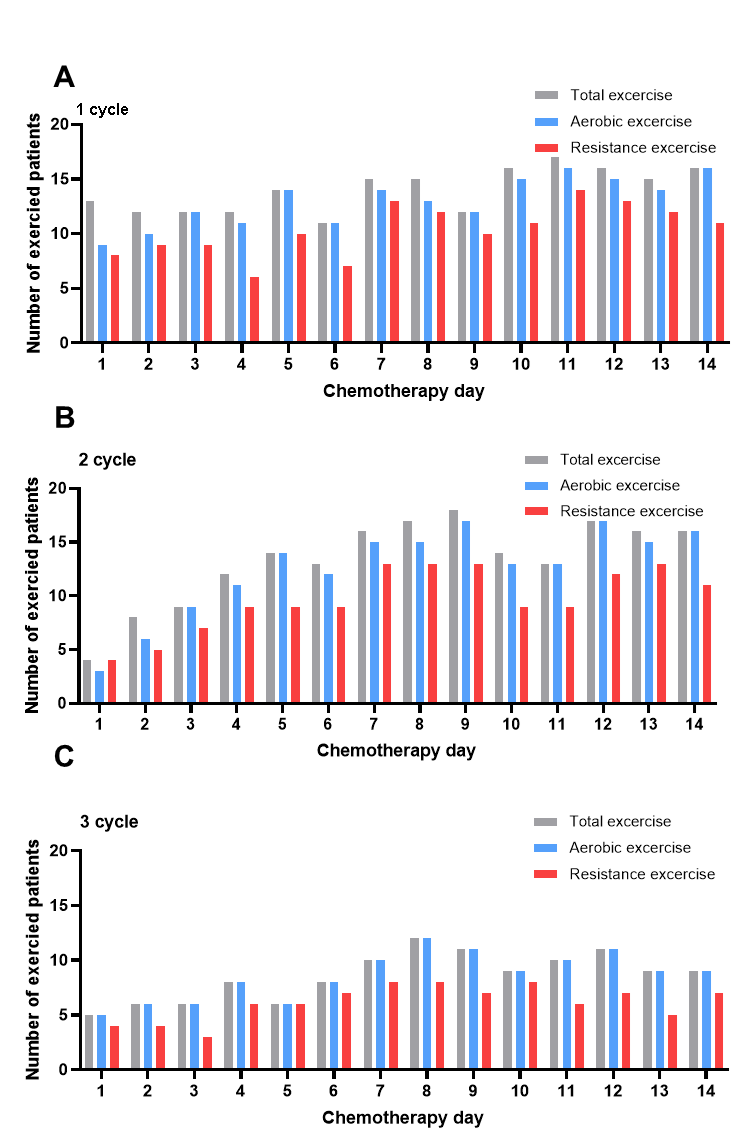

Supplement: Supplementary file 1 — Supplementary file1 (DOCX 206 KB) [file 520_2023_8017_MOESM1_ESM.docx]
