# Supplementary material for: Feasibility and safety of exercise during chemotherapy in people with gastrointestinal cancers: a pilot study
Source: Support Care Cancer. 2023 Sep 5;31(10):561. doi: 10.1007/s00520-023-08017-6 (PMC10480261; doi:10.1007/s00520-023-08017-6)
Supplement: Supplementary file 2 — Supplementary file2 (DOCX 15 KB) [file 520_2023_8017_MOESM2_ESM.docx]

**Table S1. Univariate and multivariate analyses on the overall survival.**

|  | Univariate analysis | | | Multivariate analysis | | |
| --- | --- | --- | --- | --- | --- | --- |
|  | HR | 95% CI | *p*-value | HR | 95% CI | *p*-value |
| Age |  |  |  |  |  |  |
| Age < 65 | Reference | |  |  |  |  |
| Age ≥ 65 | 1.962 | 0.396–9.725 | 0.409 | 0.369 | 0.026–5.165 | 0.459 |
| Sex |  |  |  |  |  |  |
| Male | Reference | |  |  |  |  |
| Female | 0.612 | 0.112–3.347 | 0.571 | 1.114 | 0.037–33.800 | 0.951 |
| BMI |  |  |  |  |  |  |
| ≥ 20.0 kg/m^2^ | Reference | |  |  |  |  |
| < 20.0 kg/m^2^ | 0.814 | 0.095–6.992 | 0.851 | 7.028 | 0.206–240.118 | 0.279 |
| Sarcopenia | 0.923 | 0.168–5.056 | 0.926 | 0.061 | 0.003–1.292 | 0.073 |
| Primary cancer |  |  |  |  |  |  |
| Colon cancer | Reference | |  |  |  |  |
| Other cancer | 2.946 | 0.343–25.300 | 0.325 | 29.467 | 0.594–1461.296 | 0.089 |
| Palliative chemotherapy line | |  |  |  |  |  |
| 1st line | Reference | |  |  |  |  |
| ≥ 2nd line | 1.286 | 0.150–11.009 | 0.819 | 0.676 | 0.033–13.853 | 0.800 |
| Exercise compliance |  |  |  |  |  |  |
| 6 weeks |  |  |  |  |  |  |
| Poor compliance (<50%) | Reference | |  |  |  |  |
| Good compliance (≥50%) | 0.084 | 0.010–0.726 | 0.024 | 0.002 | 0.000–0.198 | 0.008 |
| 12 weeks |  |  |  |  |  |  |
| Poor compliance (<50%) | Reference | |  |  |  |  |
| Good compliance (≥50%) | 0.020 | 0.000–17.073 | 0.257 |  |  |  |

*BMI* body mass index, *HR* hazard ratio, *CI* confidence interval.
