# Supplementary material for: Feasibility and safety of exercise during chemotherapy in people with gastrointestinal cancers: a pilot study
Source: Support Care Cancer. 2023 Sep 5;31(10):561. doi: 10.1007/s00520-023-08017-6 (PMC10480261; doi:10.1007/s00520-023-08017-6)
Supplement: Supplementary file 3 — Supplementary file3 (DOCX 14 KB) [file 520_2023_8017_MOESM3_ESM.docx]

**Table S2. Blood biomarker values and changes over exercise training**

|  | **Baseline**  **(n = 30)** | | **6 weeks**  **(n = 21)** | | | **12 weeks**  **(n = 11)** | | **Mean change over 6 weeks** | |  |
| --- | --- | --- | --- | --- | --- | --- | --- | --- | --- | --- |
| **Measure** | **Mean** | **SD** | | **Mean** | **SD** | **Mean** | **SD** | **Mean** | **95% CI** | ***p*-value** |
| Hemoglobin (g/dL) | 12.3 | 1.9 | | 11.7 | 1.5 | 11.4 | 1.8 | -0.62 | -1.49 to 0.23 | 0.146 |
| Serum albumin (g/dL) | 3.8 | 0.5 | | 4.0 | 0.4 | 3.9 | 0.5 | 0.06 | -0.15 to 0.27 | 0.555 |
| Prealbumin (mg/dL) | 18.6* | 5.1* | | 22.9 | 6.8 | 20.7 | 5.1 | 2.25 | -1.36 to 5.86 | 0.205 |
| TLC (mcL) | 1699 | 609 | | 1835 | 729 | 1899 | 932 | 49.76 | -253.87 to 353.40 | 0.736 |
| Transferrin saturation (%) | 28.5* | 15.6* | | 45 | 33.7 | 27.8 | 20.4 | 10.04 | -7.61 to 27.74 | 0.241 |
| CRP (mg/L) | 7.7 | 11.8 | | 6.9 | 15.8 | 6.8 | 12.8 | 1.09 | -5.03 to 7.21 | 0.714 |

*Missing data due to patients’ refusal (n = 3, baseline)

*CRP*C-reactive protein, *SD* standard deviation, *TLC* total leukocytes count
