# Supplementary material for: Feasibility and safety of exercise during chemotherapy in people with gastrointestinal cancers: a pilot study
Source: Support Care Cancer. 2023 Sep 5;31(10):561. doi: 10.1007/s00520-023-08017-6 (PMC10480261; doi:10.1007/s00520-023-08017-6)
Supplement: Supplementary file 4 — Supplementary file4 (DOCX 15 KB) [file 520_2023_8017_MOESM4_ESM.docx]

**Table S3. EORTC QLQ-C30 profile values and change over 6 weeks exercise training**

|  | **Baseline (n=28)** | | **6 weeks (n=21)** | | **12 weeks (n=15)** | | **Mean change** | | **P value** |
| --- | --- | --- | --- | --- | --- | --- | --- | --- | --- |
|  |  |  |  |  |  |  | **over 6 weeks** | |  |
|  | **Mean** | **SD** | **Mean** | **SD** | **Mean** | **SD** | **Mean** | **95% CI** |  |
| Global /QoL | 56.2 | 25.1 | 61.9 | 21.8 | 54.4 | 25.1 | 3.9 | 24.5 | 0.467 |
| **Functional** |  |  |  |  |  |  |  |  |  |
| Physical | 81.6 | 17.5 | 83.8 | 21.0 | 74.6 | 20.9 | 0.9 | 20.4 | 0.833 |
| Role | 83.3 | 23.5 | 79.3 | 24.0 | 75.5 | 27.3 | -1.5 | 25.7 | 0.781 |
| Emotional | 80.3 | 19.0 | 80.3 | 19.0 | 78.8 | 22.9 | 4.3 | 18.9 | 0.303 |
| Cognitive | 82.1 | 20.2 | 82.1 | 20.2 | 82.2 | 21.3 | 3.9 | 14.8 | 0.234 |
| Social | 77.9 | 21.3 | 83.3 | 24.7 | 72.2 | 30.6 | 7.1 | 32.7 | 0.329 |
| **Symptom** |  |  |  |  |  |  |  |  |  |
| Dyspnea | 17.8 | 21.2 | 11.1 | 16.1 | 13.3 | 16.9 | -4.7 | -15.7 to 6.2 | 0.379 |
| Pain | 23.8 | 25.0 | 15.8 | 23.8 | 39.2 | 28.4 | -8.7 | -21.5 to 4.1 | 0.171 |
| Fatigue | 34.9 | 26.6 | 35.9 | 24.3 | 23.3 | 30.7 | 2.1 | -9.1 to 13.3 | 0.699 |
| Insomnia | 35.7 | 38.4 | 19.0 | 27.0 | 24.4 | 26.6 | -12.6 | -24.9 to -0.4 | 0.042 |
| Appetite loss | 33.3 | 32.7 | 35.5 | 38.7 | 35.5 | 38.7 | 1.5 | -13.1 to 16.3 | 0.825 |
| Nausea and vomiting | 12.5 | 16.7 | 21.1 | 27.0 | 21.1 | 27.0 | 8.7 | -0.4 to 17.9 | 0.061 |
| Constipation | 26.1 | 31.8 | 22.2 | 34.8 | 22.2 | 34.8 | 3.1 | -12.6 to 19.0 | 0.680 |
| Diarrhea | 11.9 | 20.7 | 17.7 | 24.7 | 17.7 | 24.7 | 1.5 | -7.3 to 10.5 | 0.715 |
